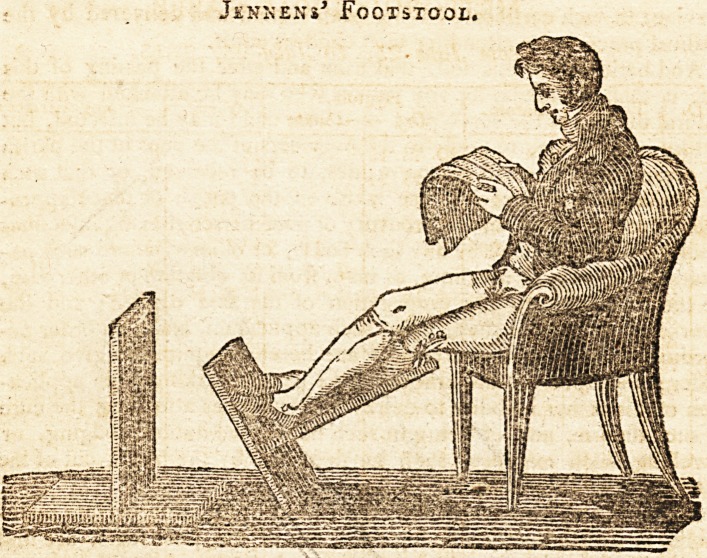# Medical and Philosophical Intelligence

**Published:** 1813-08

**Authors:** 


### MEDICAL AND PHILOSOPHICAL INTELLIGENCE.

*Copy of the amended Bill presented by Lord Boringdon to the House of Lords, entitled An Act for the more effectual Prevention of the spreading of the Infection of the Small-pox.*

WHEREAS great mortality has occurred in the last and the preceding year amongst his Majesty's subjects in the metropolis, and in many parts of the United Kingdom, from the disorders of the small-pox: and whereas from the extended, and in many cases almost universal, practice of vaccination in many parts of the world, the mortality from small-pox has in such countries altogether or in great part ceased: and whereas the Royal Colleges of Physicians and Surgeons respectively in London, and the physicians and others superintending other medical establishments, have, in authentic reports and communications, recorded their opinion as to the security afforded by vaccination against the variolous infection: and whereas it is expedient, for the security and preservation of the lives and health of his Majesty's subjects, that certain rules and regulations should be established for the giving notice of persons communicating by inoculation, or receiving by inoculation, or otherwise, the variolous infection, that precautions may be adopted against the spreading of such infection, in order that persons preferring inoculation to vaccination may resort to the same with as little danger as possible to others of his Majesty's subjects; be it therefore enacted by the King's most Excellent Majesty, by and with the advice and consent of the Lords spiritual and temporal, and Commons, in this present Parliament assembled, and by the authority of the same, that from and after the day of every person who shall inoculate with variolous matter any other person in any part of the United Kingdom, or shall be called upon as a medical practitioner to attend any patient having caught the variolous infection, shall as soon as possible, and in every case before the expiration of days, give notice thereof in writing to the clergyman or minister, and to the churchwardens and overseers of the poor in England, or two of the elders in Scotland, of the parish, township, hamlet, or place in which the person so inoculated shall reside, or if such person shall reside in any extra-parochial place, then to the clergyman, minister, churchwardens, overseers of the poor, or elders, as the case may be, of some adjoining parish or place, specifying in writing the day upon which the operation was performed, or upon which such person was called upon to attend, and the name, age, and sex, of the person so inoculated or under such infection.

And be it further enacted, that from and after the day of the parent or guardian of every child, and master of every apprentice not living with the parent or guardian, and the master or mistress of any school, and owner of every house in which any child not living with the parent (such child being under the age of years), and every infant above the age of years, and every adult respectively receiving variolous inoculation or taking variolous infection, shall, upon the day upon which such inoculation shall have taken place, or upon which it shall be ascertained that such variolous infection has been taken, or otherwise as soon after as possible, and in every case before the expiration of days, give like notice thereof in writing, or, if unable to write, then shall cause like notice to be given thereof, specifying the name and residence of the person performing the operation, and the day on which it was performed, or of the person called in to attend in case of the variolous infection being taken, and the day of such person being called in so to attend, and the name, age, and sex of the person so inoculated or under such infection as aforesaid.

And whereas it is highly expedient and necessary for the enforcing the provisions of this act, and obtaining accurate returns of the state of inoculation for the small-pox from time to time: be it therefore enacted, that from and after the passing of this act it shall not be lawful for any person to practise inoculation for the small-pox without obtaining from one of his Majesty's Royal Colleges of Physicians or Surgeons of London, Dublin, or Edinburgh, printed papers in the form in the schedule to this act annexed marked (A.), which forms shall be printed and ready for delivery by such colleges, and shall be transmitted upon application to medical practitioners for the same; and every person who shall inoculate for the small.pox shall insert the name, age, and residence of the person inoculated, and the result of the inoculation, and the churchwarden or overseer of the poor of the parish or place where such inoculation took place, with his own name and residence, upon such printed form; and at the expiration of days the person so inoculating shall transmit the said printed form, filled up with such particulars, to the registrar or secretary of the Royal College of Physicians or Surgeons from which the form was obtained; and every such return directed to the registrar or secretary of any such College of Physicians or Surgeons, (as the case may be), and marked at the top "Small-pox Return," shall go free of postage; and each of the said Royal Colleges shall transmit an annual summary or abstract of such accounts made up to the end of each year, to his Majesty's Secretary of State for the Home Department, on or before the day of in the following year.

And be it further enacted, that every medical practitioner attending a variolous patient, shall give to every such patient a certificate in writing, signed by himself, stating, that in his opinion all infection has ceased, whenever and as soon as he, to the best of his judgment, does conceive such infection to have ceased.

And be it further enacted, that no parent or guardian of any child living with the parent or guardian, or master or mistress, or owner of any house, having the care of any child not living with the parent, who shall have been inoculated or infected with the variolous disease, shall expose or permit or suffer any such child to be exposed; and no adult person shall expose himself in the public highways or streets

4 previous previous to such certificate having been signed and delivered by the medical practitioner attending such child or adult.

And be it further enacted, that from and after the passing of this act, it shall be lawful for any person who may be attacked with the natural disease of the small-pox, or whose child may be infected, but not by inoculation, to apply to the overseers of the poor of the parish or place in which such person resides, to be received, or that such child shall be received into the house in the parish or place appropriated to the reception and recovery of poor persons having infectious disorders, if there shall be any such house, or otherwise into such habitation, lodging, or dwelling, as may, from its situation or otherwise, be the least liable to the propagation of the said disease; and the overseers of the poor shall, upon such application, make an order accordingly; and the said overseers are hereby required to give such order; and upon the declaration of the person making such application of his or her inability to defray the expences attending the cure of such disease, and of being in such house, habitation, lodging, or dwelling, such expences shall be defrayed by the parish out of the rates for the relief of the poor.

And whereas in some parishes the children and other persons maintained or assisted, or receiving relief out of the poor rates, are and have been inoculated with the small-pox by order of the churchwardens, overseers, and others, (a practice which greatly contributes to perpetuate and spread this mortal contagion;) for the prevention whereof, be it further enacted, that it shall not be lawful after the passing of this act for any vestry, churchwardens, overseers, committee, or guardians, or any other persons having any controul or management of the poor in any parish, by whatever name such persons may be called, to order any person maintained or assisted by the parish, or receiving relief from the parish, or any child whose parents or parent are so maintained or assisted, or receive such relief, to be inoculated with the small-pox; and no medical or other person shall on any account whatever inoculate any such poor person for the small-pox, in pursuance of any such order as aforesaid.

SCHEDULE (A.)

| No. | Duplicate to whom granted | No. | To whom granted. | Date of Inoculation. | Duplicate Name. | Age. | Residence. | Slight Affection. | Severe Affection. | When died. |
|---|---|---|---|---|---|---|---|---|---|---|
| 1 | | 1 | | | | | | | | |

### Jennens' Footstool.

The simplicity and convenience of this footstool will generally recommend it: by the medical profession it has, however, a particular claim to be regarded as a contrivance calculated to administer ease and to facilitate the cure of those complaints in the lower extremity, in which it is important to preserve the limb in nearly a horizontal position. The mechanic arts have but seldom presented an auxiliary to medical science of more direct application, or of more effectual result. In this view it may be strongly recommended to the notice of the faculty, without the hazard of disappointing expectation.

---

A very interesting operation has recently been performed by Mr. Lynn, on a man who had lost the whole of his under lip by a cancer. The operation was conducted on the principle of a known practice in India, of restoring a lost nose, by means of the adjacent skin being raised from the flesh, and after being folded over, is made to form the part required, taking care to preserve the circulation. In this instance the skin was brought up from the throat, and the lip is so perfectly formed that the pronunciation of even the labial sounds is perfectly distinct. We have reason to believe that Mr. White, assistant surgeon to the Westminster Hospital, suggested this operation to Mr. Lynn.

---

Dr. Squire will, on Tuesday, August 17th, begin a Course of Lectures on the Theory and Practice of Midwifery, and the Diseases of Women and Children.—Particulars may be known at Dr. Squire's house, Ely-place, Holborn.

## Figures and Tables

**Figure f1:**